# Repeated loss of plastid NDH during evolution of land plants

**DOI:** 10.1093/aob/mcaf198

**Published:** 2025-09-04

**Authors:** Gitte Petersen

**Affiliations:** Department of Ecology, Environment and Plant Sciences, Stockholm University, Stockholm 106 91, Sweden

**Keywords:** Autotrophic plant, carnivorous plant, gene loss, mycoheterotrophic plant, NADH dehydrogenase-like complex, parasitic plant, photosynthesis

## Abstract

**Background:**

Advances in DNA sequencing technology have led to a rapid increase in the number of species with organelle genomes and even complete nuclear genomes being sequenced. Thousands of plastid genomes from across all major clades of land plants are now available, and one of the surprising findings is the recurring event of complete or functional loss of genes involved in cyclic electron transport during photosynthesis, i.e. the *ndh* genes that encode subunits of the chloroplast NADH dehydrogenase-like (NDH) complex. Gene loss in non-photosynthetic, heterotrophic plants might be expected, but the increasing number of losses being discovered in autotrophic plants calls into question the role and potential dispensability of the *ndh* genes and the entire NDH complex.

**Scope:**

With a focus on autotrophic plants, the present review compiles published evidence about the loss of both plastid and nuclear encoded NDH genes, providing an overview spanning all major clades of land plants. Current knowledge about the function of NDH and the possible reasons behind repeated loss are discussed.

**Conclusions:**

More than 100 independent events of plastid *ndh* gene loss have been reported from autotrophic land plants, and strong evidence exists that these losses go hand in hand with the loss of nuclear encoded NDH genes. Although loss is almost inevitable in heterotrophic plants and common among carnivorous plants, it occurs in what appears to be a random manner among normal, autotrophic plants. No single underlying reason for the events of loss can be discerned, although a link to nutrient acquisition prevails. Even in autotrophic plants, the NDH complex might simply be dispensable owing to the existence of an alternative and major pathway of cyclic electron transport around photosystem I.

## INTRODUCTION

The NADH dehydrogenase-like (NDH) complex is a protein complex composed of multiple subunits, typically encoded by a combination of 11 plastid and some 20 nuclear genes (Laughlin *et al.*, 2020; Shikanai, 2020). It has a role in photosynthesis, being part of the cyclic electron transport (CET) around photosystem I (PSI), but aspects of its function are still not entirely clear (Martín and Sabater, 2010; Strand *et al.*, 2019; Shikanai, 2020).

Since the first report of extensive loss of plastid *ndh* genes from a non-photosynthetic parasitic plant, *Epifagus virginiana* (Orobanchaceae) (Wolfe *et al.*, 1992), similar losses have been discovered in hundreds of species belonging to all major groups of land plants. The vast majority of these plants are parasites and mycoheterotrophs, and the gene loss has usually been coupled with reduced or completely lost photosynthesis (Graham *et al.*, 2017; Wicke and Naumann, 2018; Strand *et al.*, 2019). However, in 1994, a similar loss of *ndh* genes was discovered in a normal, photosynthetic gymnosperm, *Pinus thunbergii* (Pinaceae) (Wakasugi *et al.*, 1994), and since then more and more cases of loss have been reported from green, autotrophic plants.

Following advances in genome sequencing, thousands of complete plastome sequences are now available, providing a framework for studying patterns of evolution, such as *ndh* gene pseudogenization and loss (Tonti-Filippini *et al.*, 2017; Mohanta *et al.*, 2020; J. Wang *et al.*, 2024). On 27 January 2025, the number of NCBI curated plastomes (reference sequence) amounted to 14 459, and the total number of complete plastome sequences in GenBank was close to 40 000. The number of completely sequenced nuclear genomes is also increasing and, together with data from transcriptome sequencing evidence, very strongly suggests that loss of plastid *ndh* genes goes hand in hand with loss of the nuclear genes (Ruhlman *et al.*, 2015; C.-S. Lin *et al.*, 2017; Schelkunov *et al.*, 2018; Grebe *et al.*, 2019; Ke *et al.*, 2022; Schröder *et al.*, 2022; Park *et al.*, 2024*a*). Thus, the entire NDH complex appears to be missing or non-functional not only in non-photosynthetic plants, but also in a large number of autotrophs. This raises questions about the function and potential dispensability of NDH, and many are still unanswered. It has also led to much speculation about why *ndh* genes or the entire NDH complex are lost in some groups of plants, but not in others. No single reason has been found; many explanations for specific groups of plants have been suggested, and maybe losses are simply random (Jiang *et al.*, 2022).

The present review will initially describe the molecular basis and physiological function of the NDH complex and the possible consequences of its loss. Given that NDH is encoded by a combination of plastid and nuclear genes, studies providing evidence from both genomic compartments will be reviewed in order to illustrate the interrelated evolution of the genes. Genes can be either functionally lost (i.e. pseudogenized) or physically lost, but in both cases they will simply be referred to as lost, unless further detail is relevant. Subsequently, an overview of available evidence of NHD loss from all the major groups of land plants is provided. Given that the occurrence of NDH loss in parasitic and mycoheterotrophic plants is well established and has been summarized in relatively recent reviews of plastome evolution in heterotrophic plants (Graham *et al.*, 2017; Wicke and Naumann, 2018), the present review will deal only briefly with these and will focus mostly on the loss of NDH in autotrophic plants. Finally, a summary of the multiple hypotheses that have been put forwards to explain the losses will be critically reviewed, and suggestions for future directions of research will be outlined.

## THE NDH COMPLEX

The plastome of most plants includes 11 *ndh* genes (*ndhA–ndhK*) encoding subunits of the NDH complex located in the thylakoid membrane and involved in photosynthetic processes (Martín and Sabater, 2010; Strand *et al.*, 2019; Shikanai, 2020). The entire protein complex includes another 20+ nuclear encoded subunits (Laughlin *et al.*, 2020; Shikanai, 2020), with the total number of subunits being thought to have increased gradually during land plant evolution (Ruhlman *et al.*, 2015; Kato *et al.*, 2021). Here, the nuclear encoded genes are referred to as nuclear NDH genes, as opposed to the plastid *ndh* genes. The plant NDH complex is derived from the cyanobacterial ancestor of plastids and is partly homologous to mitochondrial complex I, NADH dehydrogenase, involved in respiration. Some subunits of the two complexes are homologous, whereas each complex has gained or lost other subunits in different lineages over evolutionary time (Shikanai, 2016; Laughlin *et al.*, 2019, 2020). Functionally, both complexes are responsible for initial electron transport, but whereas the mitochondrial complex I accepts electrons from NAD(P)H oxidation, the plastid NDH complex accepts electrons from reduced ferredoxin (Fd) (Laughlin *et al.*, 2020; Shikanai, 2020). Despite of the electron donor being Fd rather than NAD(P)H, the NDH complex has retained its name in order to avoid confusion (Shikanai, 2020).

As part of CET around PSI, the electrons are recycled from Fd to the plastoquinone (PQ) pool, generating proton motive force (*pmf*), thus contributing to ATP production (Shikanai, 2020). At least in angiosperms, but most probably in all streptophytes, NDH is one of two alternative pathways of CET around PSI (Hori *et al.*, 2014; Ruhlman *et al.*, 2015; Shikanai, 2020). Another and major pathway is mediated by the entirely nuclear encoded PROTON GRADIENT REGULATION 5 (PGR5) and PGR5-like Photosynthetic Phenotype 1 (PGRL1) protein complex, most likely also of cyanobacterial origin (Yamori and Shikanai, 2016; Dann and Leister, 2019; Shikanai, 2020; M. Ma *et al.*, 2021*b*).

The discovery of the two alternative pathways has led to a number of studies of their function and potential mutual redundancy (reviewed by Yamori and Shikanai, 2016; Shikanai, 2020). Studies of mutant phenotypes have shown that photosynthesis and plant growth are severely impaired in double mutants affecting both pathways, whereas single mutants can produce wild-type plants, at least in experimental growth conditions. Although the NDH complex appears to contribute far less to *pmf* formation and, consequently, to ATP production than the PGR5/PGRL1 complex, the NDH complex is suggested to play a special role in alleviating oxidative stresses in chloroplasts (Shikanai, 2020). Various environmental stresses, including extremes and rapid fluctuations in light and temperature, low CO_2_ availability, low humidity, drought and salinity, have been shown to affect NDH mutants (Horváth *et al.*, 2000; Yamori and Shikanai, 2016; Shikanai, 2020; He *et al.*, 2024). Some examples are mutant tobacco (*Nicotiana tabacum*) plants without a functional NDH complex showing retarded growth and reduced photosynthesis in low-humidity conditions (Horváth *et al.*, 2000), and similar mutant rice (*Oryza sativa*) plants having a negative biomass production and reduced photosynthesis in comparison to wild-type plants when grown at low temperature (Yamori *et al.*, 2011). Recent research shows that electron transport is particularly affected in low or fluctuating light conditions, where the NDH complex is needed for fine-tuning the electron flow (Shikanai, 2020, 2024; Q. Zhou *et al.*, 2023), and NDH might also play a special role at the onset of photosynthesis after a dark period (Basso *et al.*, 2020; Fukuda *et al.*, 2023).

In some plants using C_4_ photosynthesis, increased contents of NDH have been observed compared with the content in C_3_ plants (Strand *et al.*, 2019; M. Ma *et al.*, 2021*b*). This is most likely to be related to a higher energy demand of C_4_ plants compared with C_3_ plants, and recent evidence suggests that the NDH complex plays the primary role in electron transport in the bundle sheath cells, but not in the mesophyll cells of C_4_ plants (Ermakova *et al.*, 2024). Plants relying on crassulacean acid metabolism (CAM) photosynthesis have similar high energetic demands to C_4_ plants (Strand *et al.*, 2019), but the role of NDH in their photosynthetic pathway remains uncertain.

Owing to the evolutionary origin of NDH as homologous to the mitochondrial respiratory complex, NDH has also been hypothesized to be involved in chlororespiration, and this function is often mentioned, particularly in review papers (e.g. Daniell *et al.*, 2016; Sabater, 2021; M. Ma *et al.*, 2021*b*). However, any conclusive evidence has not been found (Peltier *et al.*, 2016; Shikanai, 2016).

## CONCOMITANT LOSS OF PLASTID AND NUCLEAR NDH GENES

In non-photosynthetic plants, loss of plastid *ndh* genes has long been known (Wolfe *et al.*, 1992) and is not surprising because the photosynthetic apparatus is not needed. However, since the first observation of pseudogenization and loss of all plastid *ndh* genes in an autotrophic plant, *Pinus thunbergii*, it has repeatedly been suggested that the genes could have been transferred to the nuclear or mitochondrial genome in order to maintain a functional NDH complex (e.g. Wakasugi *et al.*, 1994; Braukmann *et al.*, 2009; Blazier *et al.*, 2011; Jie Yu *et al.* 2022; Z.-Z. Li *et al.*, 2023). However, almost all subsequent evidence has refuted the hypothesis.

Particularly in orchids, whether with or without a normal plastid *ndh* gene complement, multiple more or less complete *ndh* genes have been discovered in the mitochondrial genome, but none of the transfers has been demonstrated as functional (C.-S. Lin *et al.*, 2015; H. T. Kim and Chase, 2017; Y.-K. Kim *et al.*, 2023). Also in Pinaceae, only fractions of *ndh* genes have been found in the mitochondria (Jie Yu *et al.*, 2022). Incorporation of plastid DNA, including long stretches with entire genes, into the mitochondrial genome of seed plants is very common, but the usual evolutionary fate of inserts is degradation, and none of the rare cases of functional transfer involves a protein-coding gene (Mower *et al.*, 2012; Sloan and Wu, 2014; X.-C. Wang *et al.*, 2018).

In an increasing number of species, from a wide variety of seed plants having lost the plastid *ndh* genes, the nuclear genome or the transcriptome has been screened for plastid genes potentially having been transferred, but no intact genes have been discovered (Ruhlman *et al.*, 2015; Sanderson *et al.*, 2015; Ranade *et al.*, 2016; Silva *et al.*, 2016; Lin *et al.*, 2017; Z. Xu *et al.*, 2018; Strand *et al.*, 2019; Folk *et al.*, 2020; Y. Sun *et al.*, 2020; Kharabian-Masouleh *et al.*, 2023; Park *et al.*, 2024*a*). This applies to members of such evolutionarily diverse plant families as the Selaginellaceae, Pinaceae, Orchidaceae, Circaeasteraceae, Papaveraceae, Saxifragaceae, Cactaceae, Simmondsiaceae and Utriculariaceae.

In plants lacking plastid *ndh* genes, further screening of nuclear genomes for the presence of nuclear encoded NDH genes has revealed that they are also lost. Absence of nuclear NDH genes has so far been reported for species of the Pinaceae and the Orchidaceae and for *Halophila ovalis* (Hydrocharitaceae) (C.-S. Lin *et al.*, 2017; Lee *et al.*, 2018). Some non-functional nuclear NDH genes were also reported in *Carnegia gigantea* (Cactaceae) (Sanderson *et al.*, 2015). Other studies including various groups of vascular plants have used either transcriptome or proteome/complexome data to search for components of the NDH complex in species lacking the plastid genes. Apart from finding products of a few nuclear NDH genes, which might have a dual function, the studies have demonstrated the lack of the NDH complex in species of Schizaeaceae, gnetophytes, Pinaceae, Orchidaceae, *Corydalis* (Papaveraceae) and *Erodium* (Geraniaceae) and in several heterotrophic plants (Ruhlman *et al.*, 2015; C.-S. Lin *et al.*, 2017; Schelkunov *et al.*, 2018; Grebe *et al.*, 2019; Ke *et al.*, 2022; Schröder *et al.*, 2022; Park *et al.*, 2024*a*). However, in *Melianthus villosus* (Francoaceae), nuclear NDH genes were found to be transcribed, but a transcription factor gene was pseudogenized (Ruhlman *et al*., 2015). Thus, most probably, the NDH complex is lacking or non-functional. The plastid *ndh* gene loss of *Melianthus* is moderate, with only four genes being affected, and thought to be relatively recent (Weng *et al.*, 2014; Ruhlman *et al.*, 2015), which might explain the retention of nuclear genes. The combined evidence from plastid and nuclear data might suggest that, at least in the case of *Melianthus*, functional loss of plastid genes precedes loss of nuclear genes.

Despite technological advances in genomic sequencing, plastome data remain far more abundant than nuclear data, and *ndh* gene loss is reported far more frequently than nuclear NDH gene loss. All current evidence suggests that loss of plastid and nuclear encoded NDH genes are concomitant events, although a screening of the nuclear genomes for NDH genes of plants possessing a normal plastid *ndh* gene complement remains to be done. However, in the event that plants should be discovered with normal, functional plastid *ndh* genes, but lacking or having malfunctioning nuclear NDH genes, it is unlikely that they would have a functional NDH protein complex, owing to the lack of most subunits. Thus, observed loss of the plastid *ndh* genes can be regarded as a very strong indication of complete loss or malfunction of the NDH complex, whereas presence of the plastid genes cannot be used to infer that the complex is functional.

## THE EXTENT OF NDH LOSS IN LAND PLANTS

**Fig. 1.**
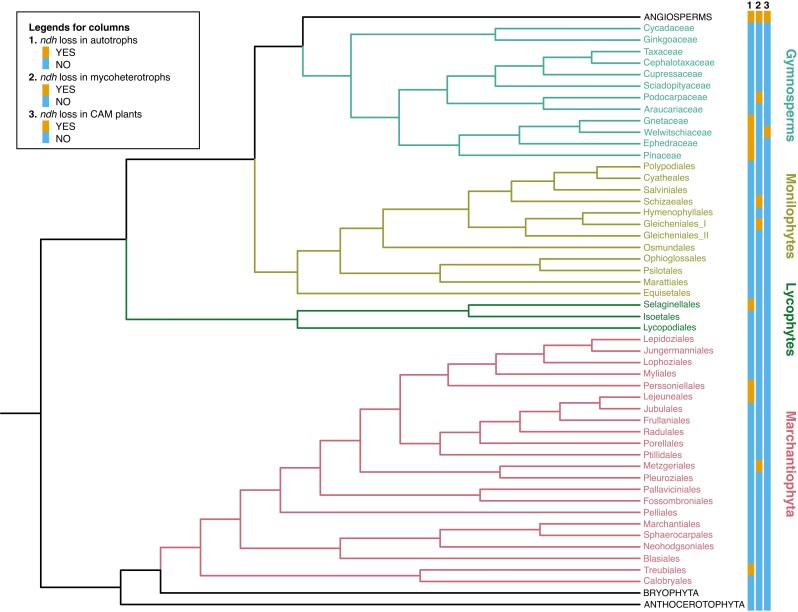
Phylogenetic distribution of *ndh* gene loss among major groups of land plants. Occurrence is indicated as YES even if it applies to only some members of a group. Dubious reports of *ndh* loss are not included (see Table 4). The phylogenetic tree is drawn according to Bechteler *et al.* (2023), Leebens-Mack *et al.* (2019), Shen *et al.* (2018) and Stull *et al.* (2021).

**Fig. 2.**
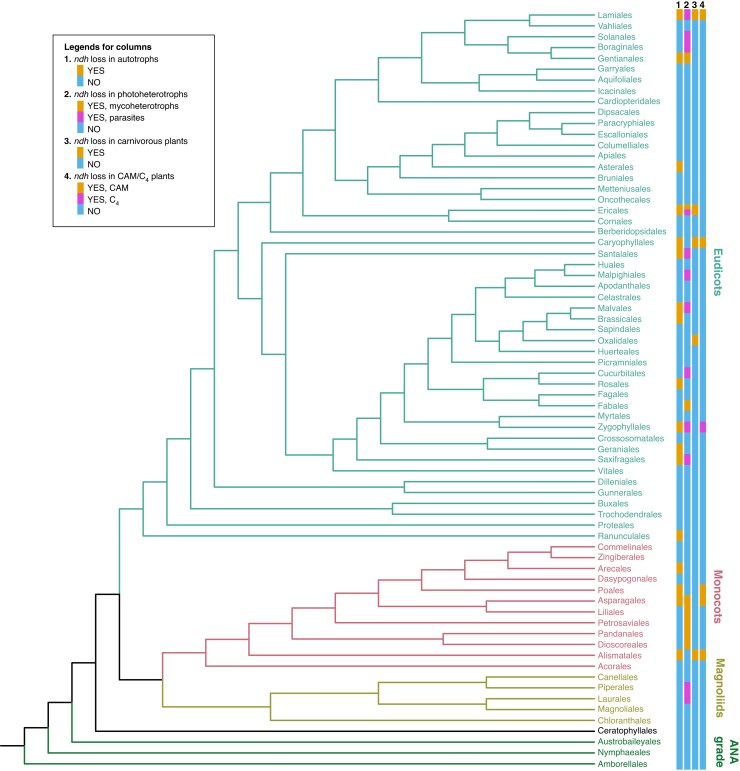
Phylogenetic distribution of *ndh* gene loss among orders of angiosperms. Dubious reports of *ndh* loss are not included (see Table 4). The phylogenetic tree is drawn according to Zuntini *et al.* (2024).

NDH loss, mostly detected through loss of plastid *ndh* genes, has occurred in all major lineages of land plants (Figs 1 and 2). The summary below deals with each of these major lineages, but three non-monophyletic groups of plants defined by their lifestyle (parasitic, mycoheterotrophic and carnivorous plants) will be covered separately owing to the frequent occurrence of gene loss, most likely coupled to the lifestyle.

The data have been compiled from literature in an attempt to provide a complete list of reported occurrences of NDH loss in all autotrophic land plants. In doing so, only studies based on complete plastome or nuclear datasets were considered, thus excluding the extensive number of phylogenetic studies using single or a few plastid loci, including partial or complete *ndhF* gene sequences. Although the latter might potentially provide additional evidence, the absence of a gene (such as *ndhF*) or annotation as a pseudogene is considered less reliable evidence. Evidence from complete plastome sequences available in GenBank that are not accompanied by peer-reviewed publications is also not considered here.

### Loss of NDH in parasitic and mycoheterotrophic plants

Extensive loss of plastid *ndh* genes was initially reported in the holoparasitic plant *Epifagus virginiana* (Wolfe *et al.*, 1992), and today the loss of *ndh* genes is seen as the first step of gene loss during degradation of plastomes in parasitic and mycoheterotrophic plants (Graham *et al.*, 2017; Wicke and Naumann, 2018). Plastome evolution in parasitic and mycoheterotrophic plants has been reviewed recently (Graham *et al.*, 2017; Wicke and Naumann, 2018), and although a large number of papers published later add evidence about loss of *ndh* genes in more taxa, the vast majority of papers do not add new evidence about the underlying mechanisms or reasons for the losses. Thus, no detailed review of *ndh* loss in parasitic and mycoheterotrophic plants will be provided here, but the phylogenetic distribution of loss is presented in Figs 1 and 2.

Given that holoparasites and full mycoheterotrophs have lost photosynthesis completely, the loss of *ndh* genes and other photosynthesis-related genes might come as no surprise, but even in hemiparasites and partial mycoheterotrophs (mixotrophs) capable of photosynthesis, the pseudogenization and loss of *ndh* genes is extremely common (Tables 1 and 2). Thus, among hemiparasitic plants only the genus *Krameria* (Krameriaceae) and a few representatives of Orobanchaceae appear to have retained an intact plastid *ndh* gene complement (Uribe-Convers *et al.*, 2014; Wicke *et al.*, 2016; Frailey *et al.*, 2018; Banerjee *et al.*, 2022; Y. Fan *et al.*, 2025). Although these data suggest that evolution of parasitism pre-dates loss of *ndh* genes, the only possible exception is found in the order Santalales, where *ndh* loss might pre-date evolution of parasitism (Edlund *et al.*, 2025).

**Table 1.** Brief overview of *ndh* gene loss in parasitic plants.

Taxonomic lineage	Hemiparasites	Holoparasites	*ndh* gene loss^1^
*Cassytha* (Lauraceae)	X	–	Yes
Hydnoraceae	–	X	Yes
Cynomoriaceae	–	X	Yes
Krameriaceae	X	–	No
Rafflesiaceae	–	X	Yes
Apodanthaceae	–	X	Yes
Cytinaceae	–	X	Yes
Santalales p.p.	X	X	Yes
Mitrastemonaceae	–	X	Yes
Lennoidoideae (Boraginaceae)	–	X	Yes
*Cuscuta* (Convolvulaceae)	X	X	Yes
Orobanchaceae p.p.	X	X	Yes, except a few hemiparasites

^1^Data from Wicke and Naumann (2018), Qu *et al.* (2019), Banerjee *et al.* (2022), Edlund *et al.* (2025); and, for Mitrastemonaceae, NC_080971.

**Table 2.** Brief overview of *ndh* gene loss in mycoheterotrophic plants.

Taxonomic lineage	Mycoheterotrophy			*ndh* gene loss^2^
	Initial^1^	Partial	Full	
*Aneura* (Aneuraceae)	–	–	X	Yes
Lycopodiaceae p.p.	X	–	–	No
Ophioglossales	X	–	–	No
Psilotales	X	–	–	No
*Stromatopteris* (Gleicheniaceae)	X	–	–	Yes
Schizaeaceae	X	–	–	Yes
*Parasitaxus* (Podocarpaceae)	––	–	X	Yes
Burmanniaceae	–	X	X	Yes, in all full and some partial
Thismiaceae	–	–	X	Yes
Triuridaceae	–	–	X	Yes
*Petrosavia* (Petrosaviaceae)	–	–	X	Yes
Corsiaceae	–	–	X	Yes
*Geosiris* (Iridaceae)	–	–	X	Yes
Orchidaceae	X	X	X	Yes, in all full, some partial, some initial
*Epirixanthes* (Polygalaceae)	–	–	X	Yes
Ericaceae p.p.	–	X	X	Yes
Gentianaceae p.p.	X	X	X	Yes

^1^Initial mycoheterotrophs include angiosperms with mycoheterotrophic seedlings and lycophytes and monilophytes with mycoheterotrophic gametophytes, but autotrophic sporophytes.

^2^Data from Graham *et al.* (2017), Lam *et al.* (2018), Wicke and Naumann (2018), Petersen *et al.* (2019), Qu *et al.* (2019) and Jing Yu *et al*. (2022).

In mycoheterotrophic plants, the correlation between loss of *ndh* genes and the degree of heterotrophy is harder to determine precisely. The physical connection between plant and fungal partners is more difficult to observe than the haustorial connection between parasitic plants and their hosts, and even the distinction between mycorrhizal and mycoheterotrophic relationships depends on the flow of organic carbon between plant and fungus (Merckx *et al.*, 2009). Furthermore, there are lineages such as orchids, where plants can be dependent on fungal partners only in the initial stages of their life cycle (initial mycoheterotrophs), and in some lycophytes and monilophytes the sporophyte generation is autotrophic, but gametophytes are mycoheterotrophic (Winther and Friedman, 2008; Graham *et al.*, 2017; Ke *et al.*, 2022; Kuo *et al.*, 2024). It is, however, clear that *ndh* loss applies to all full mycoheterotrophs and most investigated partial mycoheterotrophs (Table 2). Retention of a normal *ndh* gene complement in albino specimens of the otherwise green orchid *Epipactis helleborine* can probably be explained by a very recent transition to full mycoheterotrophy (Valencia-D *et al.*, 2021). Partial mycoheterotrophs, having retained an intact plastid *ndh* gene complement, have hitherto been found only in Orchidaceae (Feng *et al.*, 2016; Lallemand *et al.*, 2019) and, depending on the precise trophic status, possibly also in Burmanniaceae (L. Ma *et al.*, 2018). Among orchids, *ndh* loss is also observed in several, but far from all, species, which are only initial mycoheterotrophs (e.g. Y.-K. Kim *et al.*, 2020). Data from orchids are still too fragmentary to provide a complete picture of the evolution of mycoheterotrophy and *ndh* loss.

In lycophytes and monilophytes, there is no clear correlation between *ndh* loss and having mycoheterotrophic gametophytes. In monilophytes, mycoheterotrophic and usually achlorophyllous gametophytes occur in Psilotales, Ophioglossales, Schizaeaceae and *Stromatopteris* (Gleicheniaceae), but *ndh* loss has occurred only in Schizaeaceae and *Stromatopteris* (Labiak and Karol, 2017; Du *et al.*, 2022; Ke *et al.*, 2022). In lycophytes, mycoheterotrophic and achlorophyllous gametophytes are found in some, but not all, species of Lycopodiaceae (Winther and Friedman, 2008), but sequenced plastomes all appear to include a normal *ndh* gene complement (Mower *et al.*, 2019). In contrast, loss of *ndh* genes has occurred repeatedly within the heterosporic genus *Selaginella* (Selaginellaceae), with much reduced but autotrophic (macro)gametophytes (e.g. Mower *et al.*, 2019; Shim *et al.*, 2021; X.-M. Zhou *et al.*, 2022).

### Loss of NDH in carnivorous plants

A carnivorous lifestyle has evolved ≥13 times among angiosperms, but not among any other plants (Adamec *et al.*, 2021; C.-N. Fu *et al.*, 2023). Complete plastome data are available for species from nine of them, showing a complex picture of *ndh* loss and retention (Table 3). In some cases, i.e. *Triantha occidentalis* (Tofieldiaceae), Sarraceniaceae, and the monotypic genera *Cephalotus* (Cephalothaceae) and *Drosophyllum* (Drosophyllaceae), evolution of carnivory coincides with loss of *ndh* genes (M. Cao *et al.*, 2019; Baldwin *et al.*, 2023; C.-N. Fu *et al.*, 2023). In Droseraceae, all species are carnivorous and they have lost the *ndh* genes, but the evolution of carnivory probably occurred previously, in a common ancestor to Droseraceae and Nepenthaceae, the latter with all *ndh* genes intact (Gruzdev *et al.*, 2019; Nevill *et al.*, 2019; Yao *et al.*, 2019; C.-N. Fu *et al.*, 2023). In another larger clade of carnivorous plants, the Lentibulariaceae, *ndh* loss is widespread but appears to have occurred repeatedly, because several species in different clades of *Utricularia* have retained the genes (Wicke *et al.*, 2014; Silva *et al.*, 2016, 2018, 2023). Other cases of retained *ndh* genes in carnivorous plants include *Brocchinia hechtioides* (Bromeliaceae), *Roridula* (Roridulaceae) and monotypic *Triphyophyllum* (Dioncophyllaceae) (C.-N. Fu *et al.*, 2023).

**Table 3.** Brief overview of *ndh* gene loss in carnivorous plants.

Taxonomic lineage	*ndh* gene loss
*Triantha occidentalis* (Tofieldiaceae)	Yes
*Brocchinia* 2 spp. (Bromeliaceae)	No
*Catopsis berteroniana* (Bromeliaceae)	No data
*Paepalanthus bromelioides* (Eriocaulaceae)	No data
*Cephalotus folliculatus* (Cephalotaceae)	Yes
Droseraceae	Yes
*Drosophyllum lusitanicum* (Drosophyllaceae)	Yes
*Nepenthes* (Nepenthaceae)	No
*Triphyophyllum peltatum* (Dioncophyllaceae)	No
*Roridula* (Roridulaceae)	No
Sarraceniaceae	Yes
*Byblis* (Byblidaceae)	No data
Lentibulariaceae	Yes, but not in all
*Philcoxia* (Plantaginaceae)	No data

### Loss of NDH in bryophytes

Among bryophytes, loss of *ndh* genes is a rare event, so far observed only in liverworts. The best-known case is the only known mycoheterotrophic bryophyte species, *Aneura mirabilis* (Aneuraceae), a simple thalloid liverwort belonging to the order Metzgeriales (Wickett *et al.*, 2008*b*). In this species, all *ndh* genes are lost, whereas autotrophic species of *Aneura* have intact genes (Wickett *et al.*, 2008*a*). This loss of *ndh* genes in a non-photosynthetic mycoheterotrophic liverwort follows the pattern observed in other heterotrophic land plants.

In eight phylogenetically scattered, autotrophic, leafy liverworts, *ndh* gene loss has been reported (Table 4). The most extensive gene losses are observed in two species of Treubiaceae, where most genes are lost (S. Dong *et al.*, 2021; Y.-L. Xiang *et al.*, 2022). It was suggested that lost genes could have been transferred to the nuclear genome (S. Dong *et al.*, 2021), but there are no data to substantiate that. Given that nuclear transfer is not supported in any other group of land plants, it seems more likely that the genes are completely or functionally lost. In two other liverwort species, *Schistochila macrodonta* (Schistochilaceae) and *Cololejeunea lanciloba* (Lejeuneaceae), loss of a single gene (*ndhF*, reported as *nadF*) and pseudogenization of another have been reported (Y. Yu *et al.*, 2019). In four more species (see Table 4), these authors also reported a single *ndh* gene being pseudogenized. However, there is some discrepancy between the reported pseudogenes and the annotations of the GenBank sequences, and for two of the species another accession had intact genes (S. Dong *et al.*, 2021). Thus, the real extent of *ndh* gene loss in liverworts requires further investigation.

**Table 4.** List of reported loss of NDH genes in autotrophic land plants.

Major taxonomic lineage	Taxa with reported NDH gene loss	Loss of plastid *ndh* genes^1^	Loss of nuclear NDH genes^2^	References^3^
**Marchantiophyta**				
**Calobryales**				
Haplomitriaceae	*Haplomitrium blumei*	Dubious; *ndhF* reported as pseudo, but normal annotation in GenBank		(Y. Yu *et al.*, 2019)
**Treubiales**				
Treubiaceae	*Treubia lacunosa*	Multiple genes lost		(S. Dong *et al.*, 2021)
	*Apotreubia nana*	Multiple genes lost		(Y.-L. Xiang *et al.*, 2022)
**Pelliales**				
Pelliaceae	*Apopellia endiviifolia*	Dubious; *ndhD* reported as pseudo, but normal annotation in GenBank		(Y. Yu *et al.*, 2019)
**Ptilidiales**				
Ptilidiaceae	*Ptilidium pulcherrimum*	Dubious; *ndhF* reported as pseudo. Normal according to S. Dong *et al.* (2021)		(Y. Yu *et al.*, 2019)
**Radulales**				
Radulaceae	*Radula japonica*	Dubious; *ndhG* reported as pseudo. Normal according to S. Dong *et al.* (2021)		(Y. Yu *et al.*, 2019)
**Lejeuneales**				
Lejeuneaceae	*Cololejeunea lanciloba*	*ndhF* lost, *ndhD* pseudo? *ndhD* normal in GenBank		(Y. Yu *et al*., 2019)
**Perssoniellales**				
Schistochilaceae	*Schistochila macrodonta*	*ndhF* lost, *ndhG* pseudo		(Y. Yu *et al.*, 2019)
**Lycophytes**				
**Selaginellales**				
Selaginellaceae	*Selaginella* spp.	Multiple genes lost, multiple independent events. Some species normal. No functional transfer to N		(Z. Xu *et al.*, 2018; Mower *et al.*, 2019; Zhang *et al.*, 2019*a*, *b*; Shim *et al.*, 2021; Q. Xiang *et al.*, 2022; X.-M. Zhou *et al.*, 2022)
**Monilophytes**				
**Gleicheniales**				
Gleicheniaceae	*Stromatopteris moniliformis*	Multiple genes lost. Mycoheterotrophic gametophyte		(Du *et al.*, 2022)
**Schizaeales**				
Schizaeaceae	All (*Actinostachys*, *Microschizaea* and *Schizaea*)	Multiple genes lost. Mycoheterotrophic gametophytes	16 of 19 genes not transcribed. Other NDH-related genes missing	(Labiak and Karol, 2017; Ke *et al.*, 2022)
**Gymnosperms**				
**Pinales**				
Pinaceae	All (*Abies*, *Cedrus*, *Chataya*, *Keteleeria*, *Larix*, *Nothotsuga*, *Picea*, *Pinus*, *Pseudolarix*, *Pseudotsuga* and *Tsuga*)	Multiple genes lost. No functional transfer to N	10–12 of 13 investigated NDH and associated genes not transcribed or pseudo. PSI-NDH protein megacomplex missing	(Wakasugi *et al.*, 1994; Braukmann *et al.*, 2009; C.-P. Lin *et al*., 2010; C.-S. Wu *et al.*, 2011; Ruhlman *et al.*, 2015; Sudianto *et al.*, 2016; Coombe *et al.*, 2016; Yi *et al.*, 2016*a*; C.-S. Lin *et al.*, 2017; Ishizuka *et al.*, 2017, 2022; Z. Ni *et al.*, 2017; Chaw *et al.*, 2018; Liu *et al.*, 2018; Shao *et al.*, 2018, 2019, 2022, 2023; C. Guo and Xu, 2019; G.-Y. Li *et al.*, 2019; Kang *et al.*, 2019; L. Chen *et al.*, 2019; L. Su *et al.*, 2019; S. Wu *et al.*, 2019; Z.-N. Guo *et al.*, 2019; Z.-X. Fu *et al.*, 2019; S. Chen *et al.*, 2020; Y.-Y. Zhang *et al.*, 2020; J. Li *et al.*, 2021; Lubna *et al.*, 2021; N.-L. Dong *et al.*, 2021; Parmar *et al.*, 2022; Park *et al.*, 2024*b*)
**Gnetophytes**	All (*Ephedra*, *Gnetum* and *Welwitschia*)	Multiple genes lost	13 investigated NDH and associated genes not transcribed or pseudo	(C.-S. Wu *et al.*, 2007, 2009; McCoy *et al.*, 2008; Braukmann *et al.*, 2009; Ruhlman *et al.*, 2015; Chang *et al.*, 2020; Han *et al.*, 2022)
**Angiosperms**				
**Monocots**				
**Alismatales**				
Cymodoceaeae	*Amphibolis*, *Thalassodendron*	Multiple genes lost		(Iles *et al.*, 2013; Ross *et al.*, 2016; Z.-Z. Li *et al.*, 2023)
Hydrocharitaceae	subfamily Hydrilloideae (*Enhalus*, *Halophila*, *Najas*, *Nechamandra*, *Thalassia* and *Vallisneria*)	Multiple genes lost	15 genes lost in *Halophila.* Assembly and transcriptions factors also lost	(Iles *et al.*, 2013; Peredo *et al.*, 2013; Ross *et al.*, 2016; Lee *et al.*, 2018; J Chen *et al.*, 2021; Z.-Z. Li *et al.*, 2023)
Posidoniaceae	*Posidonia*	Multiple genes lost		(Iles *et al.*, 2013; Ross *et al.*, 2016)
Tofieldiaceae	*Triantha occidentalis*	Multiple genes lost. Species reported as carnivorous (Q Lin *et al.*, 2021)		(Ross *et al.*, 2016)
**Liliales**				
Liliaceae	*Lilium philadelphicum*	*ndhG* pseudo. Maybe sequencing error in multi-A region		(J.-H. Kim *et al*., 2017)
**Asparagales**				
Amaryllidaceae	*Allium paradoxum*	Multiple genes lost. All other sequenced species *Allium* normal		(Omelchenko *et al.*, 2020; Scobeyeva *et al.*, 2021)
	*Eucharis grandiflora*	Three genes lost. According to Meerow (2010) all Eucharideae have lost *ndhF* (based on PCR)		(Steele *et al.*, 2012)
	*Nerine sarniensis*	Uncertain. Maybe *ndhF* pseudo		(Könyves *et al.*, 2021)
	*Strumaria truncata*	Multiple genes lost		(Könyves *et al.*, 2021)
**Poales**				
Bromeliaceae	subfamily Hechtioideae (most; *Bakerantha* spp., *Hechtia* spp., *Mesoamerantha*)	One to multiple genes lost. Several independent events. Some species normal		(Ramírez-Morillo *et al.*, 2024)
Poaceae	*Panicum miliaceum*	Dubious; *ndhH* reported as missing. GenBank sequence misannotated. Other accessions normal		(Mohanta *et al.*, 2020)
	*Sarocalamus faberi*	Dubious; *ndhE* reported as missing. Present in GenBank sequence		(Mohanta *et al.*, 2020)
**Arecales**				
Arecaceae	*Eugeissona tristis*	Four genes lost		(Barrett *et al.*, 2016)
**Eudicots**				
**Ranunculales**				
Circaeasteraceae	*Kingdonia uniflora*	Multiple genes lost. No functional transfer to N		(Y. Sun *et al.*, 2017, 2020)
Papaveraceae	*Corydalis* spp.	One to multiple genes lost. Several independent events. Some species normal. No functional transfer to N	11 of 20 investigated genes not transcribed	(X. Xu and Wang, 2020, 2021; Raman *et al.*, 2022; S.-C. Kim *et al.*, 2023; Park *et al.*, 2024*a*)
**Saxifragales**				
Saxifragaceae	*Chrysosplenium* spp.	One to three genes independently lost in *C. carnosum* and *C. forrestii*. Other species normal		(T. Yang *et al.*, 2023)
	*Saniculiphyllum guangxiense*	Five genes lost. No functional transfer to N		(Folk *et al.*, 2020)
	*Saxifraga brunneopunctata*	Three genes lost. All (93) other species normal		(R. Yuan *et al.*, 2023)
**Geraniales**				
Francoaceae	*Melianthus villosus*	Four genes lost	NDH genes transcribed, but a nuclear transcription factor pseudo	(Weng *et al.*, 2014; Ruhlman *et al.*, 2015)
Geraniaceae	*Erodium* spp.	Multiple genes lost. Confined to one clade of *Erodium*	11 of 13 investigated NDH and associated genes not transcribed or pseudo	(Blazier *et al.*, 2011, 2016; Ruhlman *et al.*, 2015)
**Zygophyllales**				
Zygophyllaceae	subfamily Zygophylloideae (all?, *Tetraena* and *Zygophyllum*)	Multiple genes lost in *Zygophyllum* and *Tetraena*. One common event		(X. Ma *et al.*, 2019; L. Zhang *et al.*, 2021; N. Wang *et al.*, 2022; X. Wang *et al.*, 2022, 2023; Y. Yang *et al.*, 2022; Ahmad *et al.*, 2023)
**Rosales**				
Moraceae	*Ficus* spp.	*ndhF* pseudo in *F. microcarpa* and *F. vasculosa*. Independent events. Other species (22) normal		(Z.-R. Zhang *et al.*, 2022)
Ulmaceae	*Ulmus laciniata*	*ndhC* lost. Present in two other accessions in GenBank		(Zuo *et al.*, 2017)
**Oxalidales**				
Cephalotaceae	*Cephalotus follicularis*	Multiple genes lost. Carnivorous		(M. Cao *et al.*, 2019; C.-N. Fu *et al.*, 2023)
Elaeocarpaceae	*Aristotelia*, *Elaeocarpus*, *Vallea*	Dubious; *ndhK* reported lost, but appears to be an annotation error		(Y. Wang *et al.*, 2023)
**Brassicales**				
Brassicaceae	*Solms-laubachia eurycarpa*	Multiple genes lost		(X. Guo *et al.*, 2017)
Capparaceae	*Capparis spinosa* var. *herbaceae*	Multiple genes lost. *Capparis spinosa* var. *spinosa* normal		(Maurya *et al.*, 2020)
**Malvales**				
Thymelaeaceae	*Daphne kiusiana*	*ndhF* and *ndhK* pseudo		(Cho *et al.*, 2018)
**Santalales**				
Aptandraceae	All?	Multiple genes lost. The family may be parasitic, but proof is lacking		(Edlund *et al.*, 2025)
**Caryophyllales**				
Anacampserotaceae	*Anacampseros filmentosa*	Multiple genes lost		(Yao *et al.*, 2019)
Cactaceae	Subfamily Cactoideae (all?), subfamily Opuntioideae (some)	Multiple genes lost. Several independent events. No functional transfer to N	Three genes pseudo. Other genes not explored	(Sanderson *et al.*, 2015; Solórzano *et al.*, 2019; Yao *et al.*, 2019; Köhler *et al.*, 2020; 2023; Oulo *et al.*, 2020; de Almeida *et al.*, 2021; Morais da Silva *et al.*, 2021; Dalla Costa *et al.*, 2022, 2024; Qin *et al.*, 2022; Y. Ni *et al.*, 2023; Jie Yu *et al.*, 2023; Ortiz-Brunel *et al.*, 2024)
Droseraceae	All (*Aldrovanda*, *Dionaea* and *Drosera*)	Multiple genes lost. Carnivorous		(Gruzdev *et al.*, 2019; Nevill *et al.*, 2019; Yao *et al.*, 2019; C.-N. Fu *et al.*, 2023)
Drosophyllaceae	*Drosophyllum lusitanicum*	Multiple genes lost. Carnivorous		(Yao *et al.*, 2019; C.-N. Fu *et al.*, 2023)
Molluginaceae	*Pharnaceum aurantium*	Multiple genes lost		(Yao *et al.*, 2019)
Polygonaceae	*Koenigia delicatula*	Multiple gene losses. Other species normal		(D.-L. Cao *et al.*, 2022; Qu *et al.*, 2022)
Simmondsiaceae	*Simmondsia chinensis*	Multiple gene losses. No functional transfer to N		(Yao *et al.*, 2019; Kharabian-Masouleh *et al.*, 2023)
**Ericales**				
Ericaceae	*Vaccinium* spp.	*ndhF* pseudo in *V. floribundum* and *ndhG* and *ndhK* pseudo in *V. macrocarpon* cv. Stevens. According to Fahrenkrog *et al*. (2022) genes are normal but misannotated in *V. macroparpon*		(Fajardo *et al.*, 2013; López *et al.*, 2023)
Sarraceniaceae	All (*Darlingtonia*, *Heliamphora* and *Sarracenia*)	Multiple genes lost. Carnivorous		(Baldwin *et al.*, 2023; C.-N. Fu *et al.*, 2023)
**Asterales**				
Asteraceae	*Inula helianthus-aquatilis*	*ndhD* lost. Other species (4) normal		(L. Yuan *et al.*, 2025)
	*Lactuca* spp.	*ndhF* pseudo in one clade. Other species normal		(Chu *et al.*, 2022)
	*Mikania cordata*	*ndhF* pseudo. Other species (1) normal		(Y. Su *et al.*, 2018)
	*Parthenium argentatum*	Four genes pseudo. Not mentioned in the publication. Maybe sequencing/annotation errors		(Kumar *et al.*, 2009)
Campanulaceae	*Adenophora remotiflora*	*ndhB* pseudo		(K.-A. Kim *et al.*, 2016)
	*Downingia*, *Legenere*, *Porterella*	Multiple genes lost. One common event		(Knox and Li, 2017)
	*Isotoma hypocrateriformis*	Multiple genes lost		(Knox and Li, 2017)
	*Trachelium caeruleum*	Dubious; *ndhK* pseudo, but annotation error according to Cheon *et al.* (2017)		(Haberle *et al.*, 2008)
**Dipsacales**				
Adoxaceae	*Adoxa*, *Sambucus*, *Sinadoxa*, *Tetradoxa*, *Viburnum*	Error; *ndhF* reported missing in six species in five genera, but appear to be missed owing to an inversion		(W.-B. Fan *et al.*, 2018)
**Gentianales**				
Gentianaceae	*Gentiana* spp., *Sinogentiana*	Multiple genes lost. Multiple independent events		(P.-C. Fu *et al.*, 2016, 2021, 2022; S.-S. Sun *et al.*, 2018, 2024; Ya *et al.*, 2020; Ya and Miao, 2021; Jing Yu *et al.*, 2022)
Rubiaceae	*Cruckshanksia pumila*	Multiple genes lost		(Thureborn *et al.*, 2024)
**Lamiales**				
Acanthaceae	*Acanthus mollis*	Five genes lost. Other species (2) normal		(D. Ma *et al.*, 2023)
Plantaginaceae	*Littorella* (*Plantago*) *uniflora*	Multiple genes lost. *Plantago* spp. otherwise normal		(Mower *et al.*, 2021)
Lentibulariaceae	*Genlisea*, *Pinguicula*, *Utricularia* spp.	Multiple genes lost. Multiple independent losses. Some species of *Utricularia* normal. No functional transfer to N		(Wicke *et al.*, 2014; Silva *et al.*, 2016, 2018, 2023; C.-N. Fu *et al.*, 2023)

^1^Details of individual genes or numbers are given only if fewer than half the genes (five) are lost. Unless details of pseudogenes are listed, loss equals functional loss (physical loss + pseudogenization).

^2^Few studies using transcriptome or genome data exist, and they explore variable numbers of genes.

^3^Only papers specifically addressing NDH gene loss are listed except if cited by others for gene loss. The cited studies related to plastid data include complete plastome sequences or, more rarely, other complete or nearly complete plastid gene overviews.

### Loss of NDH in lycophytes

In lycophytes, loss of *ndh* genes is restricted to the genus *Selaginella*, but it does not apply to all species. The first report was for the species *Selaginella tamariscina*, in which all genes are lost from the plastome, and nuclear data showed no evidence of transfer (Z. Xu *et al.*, 2018). Subsequent studies have revealed multiple independent losses and variations in the numbers of intact and functionally or completely lost genes (Mower *et al.*, 2019; Zhang *et al.*, 2019*a*; Shim *et al*., 2021; X.-M. Zhou *et al.*, 2022). At the same time, a number of species in *Selaginella* have an alternative structure of the plastome, with direct repeats instead of inverted repeats or lacking long repeats entirely (Mower *et al.*, 2019; Zhang *et al*., 2019*a*, *b*; Q. Xiang *et al.*, 2022; X.-M. Zhou *et al.*, 2022). Loss of *ndh* genes appears to be more common among species with an alternative repeat structure, but it is not restricted to those.

Some *Selaginella* species are confined to dry habitats and tolerate considerable desiccation, viz. ‘resurrection’ plants. In a less drought-tolerant species with intact *ndh* genes, *S. moellendorfii*, it was demonstrated that the genes were predominantly expressed in the hydrated state of the plant (Z. Xu *et al.*, 2018). Coupled with the absence of *ndh* genes in drought-tolerant *S. tamariscina*, the authors suggested a link between *ndh* gene loss and dehydration/resurrection. With more species investigated, further evidence was provided for a link between drought tolerance and *ndh* loss (Zhang *et al.*, 2019*b*; X.-M. Zhou *et al.*, 2022), but not all species fit the pattern, and no correlation analyses have been made.

### Loss of NDH in monilophytes

Among the monilophytes, loss of *ndh* genes is known only from a few fern species. In Schizaeaceae, all investigated species lack all *ndh* genes (Labiak and Karol, 2017; Ke *et al.*, 2022), and a concomitant loss of the nuclear encoded NDH genes is suggested based on transcriptome sequencing (Ke *et al.*, 2022). The only other known loss involves *Stromatopteris moniliformis* (Gleicheniaceae), in which eight of the *ndh* genes are reported as missing (Du *et al.*, 2022).

Both *Stromatopteris* and Schizaeaceae have autotrophic sporophytes, but they produce gametophytes having a mycoheterotrophic lifestyle, thus the loss of *ndh* genes might be associated with mycoheterotrophy (Ke *et al.*, 2022). Although some Schizaeaceae gametophytes are chlorophyllous, all are mycoheterotrophic, and the evolution of mycoheterotrophy within Schizaeales coincides with the loss of *ndh* genes (Ke *et al.*, 2022). However, there is no strict correlation between mycoheterotrophic gametophytes and *ndh* gene loss in monilophytes. Ophioglossaceae and Psilotaceae also have mycoheterotrophic gametophytes (Merckx and Freudenstein, 2010) but intact *ndh* genes (Du *et al.*, 2022; Kuo *et al.*, 2024). Given that *ndh* gene loss has been reported only for monilophytes with mycoheterotrophic gametophytes, mycoheterotrophy might be a prerequisite for the loss.

### Loss of NDH in gymnosperms

Three lineages of gymnosperms are characterized by loss of *ndh* genes: the gnetophytes, the Pinaceae and the monotypic genus *Parasitaxus* (Podocarpaceae). Since the reported functional loss of all *ndh* genes from the plastome of *Pinus thunbergii* (Wakasugi *et al.*, 1994), subsequent studies have demonstrated that loss of *ndh* genes applies to all investigated species in Pinaceae (e.g. C.-P. Lin *et al.*, 2010; Sudianto *et al.*, 2016; Z. Ni *et al.*, 2017; Chaw *et al.*, 2018; Lubna *et al*., 2021). Several sources of data, primarily from *Picea abies*, refute the hypothesis of functional gene transfer and demonstrate lack of the nuclear NDH genes and the entire protein complex (Ruhlman *et al.*, 2015; Ranade *et al.*, 2016; C.-S. Lin *et al.*, 2017; Grebe *et al.*, 2019). Among gnetophytes (*Ephedra*, *Gnetum* and *Welwitschia*), all investigated species have lost the *ndh* genes completely, or a few might remain as pseudogenes (C.-S. Wu *et al.*, 2007, 2009; McCoy *et al.*, 2008; Braukmann *et al.*, 2009; Han *et al.*, 2022). As for Pinaceae, a concomitant loss of nuclear NDH genes has been documented for representatives of all three gnetophyte genera (Ruhlman *et al.*, 2015). The loss of NDH in gnetophytes and Pinaceae might be one common, ancestral event if the two lineages are sister groups (Fig. 1). However, this relationship is still uncertain (Leebens-Mack *et al.*, 2019; Stull *et al.*, 2021). Finally, all *ndh* genes are lost in the only heterotrophic gymnosperm, *Parasitaxus usta* (Qu *et al.*, 2019). Owing to the non-photosynthetic habit of this species, the loss is unsurprising.

Many gymnosperms have a modified plastome structure lacking inverted repeats or having only very short and potentially novel repeat regions (e.g. C.-P. Lin *et al.*, 2010; Chaw *et al.*, 2018; Lubna *et al.*, 2021). However, the loss of *ndh* genes appears not to be linked strictly to structural modifications (see below).

### Loss of NDH in autotrophic angiosperms

With an increasing number of complete angiosperm plastomes being sequenced, the number of losses of *ndh* genes being reported continuously increases. Excluding parasitic and mycoheterotrophic plants, publications currently describe >80 independent events of *ndh* loss in 40 families in 20 orders of autotrophic angiosperms (Table 4). This number of independent events of loss might be biased, e.g. by phylogenetic ambiguity, in particular within genera, by lack of formal ancestral character state reconstruction, and by erroneous reports (see notes in Table 4). With additional plastome data becoming available, the real number of independent events of *ndh* gene loss will, most likely, be considerably higher. Although the known losses of *ndh* genes occur in most parts of the angiosperm phylogeny, none is yet reported from the autotrophic species of the Amborellales, Nymphaeales, Austrobaileyales grade or the magnoliids. Among the autotrophic monocots, *ndh* loss has occurred in at least four orders and seven families. In Alismatales, where four events of loss are confined to four different families (Cymodoceaceae, Hydrocharitaceae, Posidoniaceae and Tofieldiaceae), it is noteworthy that three of these groups include marine aquatics (Ross *et al.*, 2016; Z.-Z. Li *et al.*, 2023). However, not all marine aquatics have lost *ndh* genes, and *ndh* loss is also found in some freshwater aquatics and in one species of *Triantha* (Tofieldiaceae) now recognized as a carnivorous plant (Q. Lin *et al.*, 2021). For one marine aquatic species, *Halophila ovalis* (Hydrocharitaceae), the loss of plastid *ndh* genes is accompanied by the loss of nuclear NDH genes (Lee *et al.*, 2018).

Another example of *ndh* loss in the monocots is found in Bromeliaceae subfamily Hechtioideae. Most, but not all, investigated species in this subfamily have loss of one or more genes, and a possible link between *ndh* loss and CAM photosynthesis was suggested (Ramírez-Morillo *et al.*, 2024). The Bromeliaceae also include a few carnivorous species assigned to other subfamilies, but the one with a complete plastome sequenced has intact *ndh* genes (C.-N. Fu *et al.*, 2023); see above for further discussion about carnivorous plants and *ndh* loss. In other autotrophic monocots, loss of *ndh* genes seems rare, with a few scattered cases in the Amaryllidaceae and Arecaceae (Table 4).

Among autotrophic eudicots, *ndh* loss has been reported in 30 families in 14 orders. The loss of *ndh* genes in one clade of the genus *Erodium* (Geraniaceae) represents the first reported loss in autotrophic angiosperms (Blazier *et al.*, 2011, 2016). A subsequent transcriptome study showed that both plastid and nuclear NDH genes were missing (Ruhlman *et al.*, 2015). Another loss of *ndh* genes in the Geraniales has been found in *Melianthus villosus* (Francoaceae) (Weng *et al.*, 2014).

The largest clade of autotrophic angiosperm species having lost *ndh* genes is probably found in the Cactaceae (Caryophyllales). Gene loss was first reported in *Carnegia gigantea* (Sanderson *et al.*, 2015), and a number of subsequent studies have demonstrated that *ndh* loss most probably applies to the entire subfamily Cactoideae (Solórzano *et al.*, 2019; Oulo *et al.*, 2020; Amaral *et al.*, 2021; de Almeida *et al.*, 2021; Morais da Silva *et al.*, 2021; Dalla Costa *et al.*, 2022, 2024; Qin *et al.*, 2022; Jie Yu *et al.*, 2023; Ortiz-Brunel *et al.*, 2024). Additional independent events of *ndh* gene loss were found in the subfamily Opuntioideae (Köhler *et al.*, 2020, 2023). In Cactaceae, nuclear data from *Carnegia* suggest complete lack or non-functioning of the entire NDH complex (Sanderson *et al.*, 2015). In *Carnegia*, a link between loss of *ndh* genes and loss of the inverted repeat (IR) was discussed (Sanderson *et al.*, 2015), but later evidence does not show a clear association between structural changes, including IR loss, and *ndh* loss (Köhler *et al.*, 2023; Jie Yu *et al.*, 2023). Instead, a link between *ndh* loss and CAM photosynthesis was suggested (Köhler *et al.*, 2020). In the Caryophyllales, several other *ndh* gene losses are reported (Table 4). In monotypic *Simmondsia* (Simmondsiaceae), a search for the missing plastid gene in the nuclear genome recovered only a fragment of one gene (Kharabian-Masouleh *et al.*, 2023).

In a few distantly related genera of angiosperms, multiple independent losses of *ndh* genes are reported. In the genus *Corydalis* (Ranunculales: Papaveraceae), roughly half of the sequenced species have lost one or more *ndh* genes, and current phylogenetic evidence strongly suggests multiple independent events of loss (X. Xu and Wang, 2021; Raman *et al.*, 2022; S.-C. Kim *et al.*, 2023). A transcriptome study of one species showed no presence of plastid *ndh* genes, and most nuclear NDH genes were also missing (Park *et al.*, 2024*a*). Thus, *Corydalis* might be an excellent group for studying the early evolutionary steps towards NDH loss and the association between functional loss of plastid and nuclear genes. In another species from the Ranunculales, *Kingdonia uniflora* (Circaeasteraceae), in which *ndh* genes are also lost, a screening of the nuclear genome did not reveal any presence of transferred genes (Y. Sun *et al.*, 2017 , 2020).

*Gentiana* (Gentianaceae) provides another example of a genus with repeated *ndh* loss. Phylogenetic evidence suggests four independent events of loss in *Gentiana* plus another one within the closely related genus *Sinogentiana* (P.-C. Fu *et al.*, 2021). Although an earlier study of a few species tried to link loss to environmental factors (P.-C. Fu *et al.*, 2016), no clear pattern was found in a more comprehensive study (P.-C. Fu *et al.*, 2021). However, what might remain to be investigated is the mycorrhizal associations and a possible link to the loss of *ndh* genes. Mycoheterotrophic seedling growth was found in *Gentiana zollingeri*, a species in which *ndh* genes are lost (Jing Yu *et al.*, 2022), and could be more widespread in the genus and other relatives (Yamato *et al.*, 2021). Full or partial mycoheterotrophy has evolved repeatedly in Gentianaceae and is associated with *ndh* loss (Graham *et al.*, 2017; Lam *et al.*, 2018), but the occurrence of initial mycoheterotrophy is mostly unexplored.

Among the remaining cases of eudicot *ndh* loss, several involve a single or few species from a clade, e.g. a genus, where other hitherto investigated species have intact *ndh* genes. Some examples are *Chrysosplenium* (Saxifragaceae), *Saxifraga* (Saxifragaceae), *Ficus* (Moraceae) and *Inula* (Asteraceae) (Z.-R. Zhang *et al.*, 2022; T. Yang *et al.*, 2023; R. Yuan *et al.*, 2023; L. Yuan *et al.*, 2025). The gene losses reported in these genera typically involve one or a few genes, consistent with recent evolutionary events. *Capparis spinosa* (Capparaceae) even provides an example of infraspecific variation, with one variety showing extensive *ndh* gene loss, whereas another variety and other species have intact genes (Maurya *et al.*, 2020).

## EXPLANATIONS OF NDH LOSS

Given that the function of the NDH complex is not yet completely understood, several hypotheses attempting to explain the observed losses have been put forwards. The explanations typically fall into different categories, albeit sometimes overlapping, and they usually reflect whatever possible atypical trait might be assigned to the plants exhibiting NDH loss.

### Reduced or lost photosynthesis

Generally, loss of NDH has been associated with loss of photosynthetic capacity in holoparasites and fully mycoheterotrophic plants, and with potentially reduced need for photosynthesis in hemiparasites and partial mycoheterotrophs (Graham *et al.*, 2017; Wicke and Naumann, 2018; Strand *et al.*, 2019). Given the involvement of NDH in the photosynthetic process, this is intuitively a plausible explanation, but it cannot explain the increasing amount of evidence for loss in autotrophic plants. As mentioned above, it is also not entirely clear whether the order of events is always the same: evolution of parasitism first or loss of NDH first. If a parasitic or mycoheterotrophic habit and reduced need for photosynthesis evolves first, then gradual loss of *ndh* and other photosynthesis genes would be a natural route of plastome modification (e.g. Barrett and Davis, 2012; Wicke *et al.*, 2016; Graham *et al.*, 2017), but if NDH loss can precede evolution of parasitism, it could even be a driving force towards evolution of a parasitic habit (Edlund *et al.*, 2025). The latter possibility might apply to the Santalales, but phylogenetic ambiguity and a lack of data about the autotrophic or heterotrophic habit of some crucial taxa prevent conclusive reconstruction of the ancestral character states (Edlund *et al.*, 2025).

### Nutrient acquisition

An increasing number of carnivorous plants are also being discovered to lack *ndh* genes and, most probably, a functional NDH complex (Wicke *et al.*, 2014; Ross *et al.*, 2016; Silva *et al.*, 2016, 2018; M. Cao *et al.*, 2019; Gruzdev *et al.*, 2019; Nevill *et al.*, 2019; Q. Lin *et al.*, 2021; Baldwin *et al.*, 2023; C.-N. Fu *et al.*, 2023), albeit not as consistently as in parasitic and mycoheterotrophic plants (see above section on carnivorous plants). The loss of *ndh* genes in some carnivorous lineages has been associated with the acquisition of organic nutrients through prey, comparable to the acquisition of nutrients in parasitic and mycoheterotrophic plants, and several authors have suggested a general correlation between NDH loss and substrates or nutrient sources of plants (Wicke *et al.*, 2011; Ross *et al.*, 2016; Tonti-Filippini *et al.*, 2017; Nevill *et al.*, 2019; Strand *et al.*, 2019; Baldwin *et al.*, 2023; C.-N. Fu *et al.*, 2023). However, in orchids where both NDH losses and nutritional mode shifts have occurred repeatedly, little, if any, evidence exists for a correlation (Lallemand *et al.*, 2019; Strand *et al.*, 2019). Nor was NDH loss correlated with the choice of terrestrial, epiphytic or lithophytic habit in orchids that are initial mycoheterotrophs (Strand *et al.*, 2019).

Among nutrients, particular attention has been paid to nitrogen. An explanation for a potential correlation with NDH loss was based partly on the observation that another pathway of cyclic electron transport, other than the major PGR5/PGRL1 pathway, is crucial in conditions of nitrogen deprivation in *Chlamydomonas reinhardtii* chloroplasts (Saroussi *et al.*, 2016). Thus, provided that the NDH pathway plays similar roles in *Chlamydomonas* and land plants, it was hypothesized that NDH could be dispensable in plants using an alternative nitrogen source (Tonti-Filippini *et al.*, 2017). However, *Chlamydomonas* and most other non-streptophyte algae have lost the NDH pathway of cyclic electron transport, but are instead able to target a group of single-subunit flavoenzymes (NDH-2), also used in mitochondrial respiration, to the chloroplast (Peltier *et al.*, 2016; Nikkanen *et al.*, 2021). The NDH and NDH-2 pathways are unrelated evolutionarily, and assuming a similar effect of nitrogen deprivation or source is highly speculative. However, as originally suggested by Peltier *et al.* (2016), it remains of interest to determine whether the loss of a functional NDH pathway in streptophytes, including land plants, is also associated with the targeting of NDH-2 enzymes to the chloroplast.

Furthermore, it has been proposed that acquisition of organic nitrogen rather than inorganic nitrate might render NDH superfluous if the reductant derived from photosynthesis required for nitrate assimilation is associated with the function of NDH (Nevill *et al.*, 2019). The authors noted that this could potentially even explain NDH loss in fully autotrophic plants relying heavily on mycorrhizal associations, such as the Pinaceae. The idea of a potential connection between the loss of NDH and mycorrhizal associations was proposed earlier by Wicke *et al.* (2011), although noting that the events of gene loss and presence of mycorrhiza appeared imperfectly correlated.

Prompted by observed *ndh* gene losses in some submerged aquatic species of Alismatales, a connection between NDH loss, aquatic habit and levels of leaf nitrogen was also suggested (Ross *et al.*, 2016). It was proposed that reduced photosynthesis and limited nutrient availability in a submerged habitat could lead to reduced investment in leaf nitrogen and favour NDH loss (Ross *et al.*, 2016). In a marine,alismatid species, *Halophila ovalis*, lacking the NDH complex, two protein complexes involved in the uptake of nitrate are also missing, but whether this is connected to the loss of NDH is uncertain (Lee *et al.*, 2018).

### CAM and C_4_ photosynthesis

As mentioned above, an increased importance of the NDH complex in plants using C_4_ or CAM photosynthesis might be hypothesized (Strand *et al.*, 2019; Shikanai, 2020; M. Ma *et al.*, 2021*a*). However, instances of NDH loss among CAM plants can be observed, i.e. in *Welwitschia*, Bromeliaceae subfam. Hechtioideae, most Cactaceae, *Littorella* (Plantaginaceae), *Vallisneria* (Hydrocharitaceae) and several Orchidaceae (for reviews of CAM plants, see Keeley, 1998; Keeley and Rundel, 2003; Silvera *et al.*, 2010), and some authors have suggested a connection (Köhler *et al.*, 2020; Mower *et al.*, 2021; Ramírez-Morillo *et al.*, 2024). Although many plant species have not been screened for the use of photosynthetic pathways or for NDH loss, it seems that there is no general correlation. Strand *et al.* (2019) disputed an association among orchids, and although the majority of all cacti use CAM photosynthesis, not all of them have lost NDH (Sanderson *et al.*, 2015; Solórzano *et al.*, 2019; Yao *et al.*, 2019; Köhler *et al.*, 2020; Morais da Silva *et al.*, 2021). Among the gnetophytes, where all members have lost NDH, only *Welwitschia* uses CAM photosynthesis. Thus, for CAM photosynthesis the NDH complex appears not to be essential, but not all CAM plants lose NDH.

Plants using C_4_ photosynthesis are apparently more prone to retain the NDH complex. Among all the lineages of C_4_ plants (Sage, 2016), *Tetraena simplex* (Zygophyllaceae) is hitherto the only one for which loss of *ndh* genes is reported (Ahmad *et al.*, 2023). Loss of *ndh* genes seems to have occurred in the common ancestor to a clade including *Zygophyllum* and *Tetraena* (L. Zhang *et al.*, 2021; X. Wang *et al.*, 2022; Y. Yang *et al.*, 2022), but *T. simplex* is the only species with C_4_ photosynthesis (Lauterbach *et al*, 2016). Thus, the development of C_4_ photosynthesis is possible even in plants presumably lacking the NDH complex. The monocot aquatic, *Hydrilla verticallata* (Hydrocharitaceae), is likely to be another example of a C_4_ plant lacking NDH. Although plastid or nuclear data are not yet available for *Hydrilla*, its phylogenetic position within an *ndh*-lacking clade of Hydrocharitaceae, the subfamily Hydrilloideae (Petersen *et al.*, 2016; Ross *et al.*, 2016), strongly suggests the absence of a functional NDH complex in *H. verticillata*. This will be another case of C_4_ development within a clade already having lost the NDH complex. Notably, *H. verticillata* and *Tetraena simplex* represent both the most common type of C_4_ plant anatomy, the dual-cell Kranz type (*Tetraena*), and the more rare single-cell type (*Hydrilla*) (Crookston and Moss, 1972; Bowes, 2011; von Caemmerer *et al.*, 2014). The latter type is shared with another Hydrocharitaceae aquatic, *Elodea densa* (syn. *Egeria densa*), which, unfortunately, also lacks molecular data. However, two other species of *Elodea* have data showing a normal *ndh* gene complement (Ross *et al.*, 2016), suggesting that *ndh* gene loss and single-cell C_4_ photosynthesis in Hydrocharitaceae are not correlated. Also terrestrial, single-cell C_4_ species in Amaranthaceae have standard plastomes (Sharpe *et al.*, 2020).

### Aquatic habit

Within the Alismatales, plastid *ndh* genes have most probably been lost four times, three of which are in purely aquatic lineages, and the only loss in a terrestrial species involves the carnivorous *Triantha occidentalis* (Ross *et al.*, 2016; Lee *et al.*, 2018; Q. Lin *et al.*, 2021). Early on, it was suggested that reduced light stress in aquatic habitats could render NDH superfluous in submerged alismatids (Iles *et al.*, 2013; Peredo *et al.*, 2013). The Alismatales include a large number of freshwater aquatics and the only marine angiosperms. Loss of *ndh* genes has been found in both marine and freshwater lineages, but other species from these environments have normal *ndh* gene complements. Although *ndh* loss is found mostly in submerged species, no clear correlation between habit or habitat can be observed (Ross *et al.*, 2016). A few other autotrophic, semiaquatic plants (*Saniculiphyllum* in Saxifragaceae, *Littorella* in Plantaginaceae) have also lost some or all *ndh* genes, but many other sequenced angiosperm aquatics have retained the genes (Folk *et al.*, 2020; Mower *et al.*, 2021). In a recent paper, some semiaquatic species of Podostemaceae are also cited as having experienced *ndh* gene loss (Folk *et al.*, 2020), but the presence of *ndhH* pseudogenes is caused by location at the IR border, thus another copy is complete, and no functional loss has been observed within the family (Bedoya *et al*., 2019).

In Lentibulariaceae, including both aquatic and terrestrial carnivorous species, Silva *et al.* (2016 , 2018) originally suggested a correlation between loss of *ndh* genes and a terrestrial habit, but a more thorough taxon sampling has shown that this is not the case (Silva *et al.*, 2023).

### Other environmental factors

As an ever-increasing number of *ndh* gene losses are reported from seed plants (Table 4), it becomes increasingly difficult to envision a single common cause for *ndh* gene loss and what is most likely complete loss of the NDH complex. Given that numerous environmental stress factors have been shown to affect NDH mutants (see e.g. Yamori and Shikanai, 2016; Shikanai, 2020), it is often assumed that abiotic stress will cause retention of the genes, whereas they are dispensable in less stressful conditions (Ruhlman *et al.*, 2015). Many, often contradictory, speculations have been made about possible abiotic stresses that could have caused retention of the NDH complex in some species compared with loss in others. For example, the NDH loss in some aquatic alismatids mentioned above was suggested to be related to low light stress in aquatic environments (Iles *et al.*, 2013; Peredo *et al.*, 2013), whereas it was proposed that NDH could be dispensable in terrestrial Lentibulariaceae owing to this environment being less stressful than the aquatic one (Silva *et al.*, 2018).

Several lineages contain species with normal gene complements, in addition to others lacking a functional *ndh* gene complement. The difference exists even within genera, such as e.g. *Allium* (Amaryllidaceae) (Omelchenko *et al.*, 2020; Scobeyeva *et al.*, 2021), *Gentiana* (P.-C. Fu *et al.*, 2016, 2021; S.-S. Sun *et al.*, 2018), *Koenigia* (Polygonaceae) (Qu *et al.*, 2022), *Erodium* (Blazier *et al.*, 2011) and *Utricularia* (Silva *et al.*, 2023), and within varieties of *Capparis spinosa* (Maurya *et al.*, 2020). In *Gentiana*, where loss has occurred repeatedly, P.-C. Fu *et al.* (2016) initially suggested that a cold habitat would be less stress-inducing than a warmer one, thus promoting *ndh* gene loss in cold, high-elevation environments. However, with a dense taxon sampling they found no correlation between current habitat choice and *ndh* loss, although they suggested that *ndh* loss might still be associated with historical climate changes (Fu *et al.*, 2021). In *Allium*, only one of multiple investigated species has lost *ndh* genes (*Allium paradoxum*), and the loss was suggested to be associated with a shaded environment (Omelchenko *et al.*, 2020), whereas retention and positive selection of *ndh* genes in other species were suggested to be associated with abiotic factors of high elevations (Scobeyeva *et al.*, 2021); in contrast to the explanatory scenarios proposed for *Gentiana*. Also in *Koenigia*, *ndh* loss has so far been observed in only one of several species (*K. delicatula*), and the loss is associated with increased substitution rates of organelle genes (Qu *et al.*, 2022). The authors speculated both about a connection between *ndh* loss and dwarf habit of the species, and about how historical climate changes (in this case, global warming) at the time of species diversification could possibly be related to the evolutionary trait divergence between *K. delicatula* and other species of *Koenigia*. However, *K. delicatula* is not the only dwarf species in the genus, or the only one exhibiting accelerated substitution rates, thus, few, if any, data support the ideas.

The above explanations for NDH loss are only a few examples from the many occurring in the literature. Although well-argued hypotheses of NDH loss exist, a wide range of papers try to explain an observed loss with almost any trait that might distinguish plants with and without a functional NDH complex.

### Plastome alterations

In heterotrophic plants, relaxed purifying selection and increased substitution rates lead to functional or complete gene loss of non-essential plastome genes. The *ndh* genes are the first to be lost, but eventually the majority or even all plastid genes might be lost, and the plastome might be highly rearranged (Wicke *et al.*, 2016; Graham *et al.*, 2017; Wicke and Naumann, 2018). In autotrophic plants, also having lost *ndh* genes, additional gene losses are generally few, and no other loss of specific plastome genes appears to be associated consistently with loss of *ndh*.

Only few studies of autotrophic plants provide evidence of potential changes to the mutation rates or selection pressure on *ndh* genes in clades where loss has occurred. In some cases, such as *Selaginella*, *Saniculiphyllum* and *Littorella*, *ndh* loss is not correlated with a generally increased mutation rate of plastid genes (Mower *et al.*, 2019, 2021; Folk *et al.*, 2020, Q. Xiang *et al*., 2022). However, in other cases, such as *Erodium* and *Allium*, a positive association has been found (Blazier *et al.*, 2016; Scobeyeva *et al.*, 2021). In *Saniculiphyllum*, tests revealed no statistically significant changes to the selection regime of the *ndh* genes (Folk *et al.*, 2020), and data from *Allium* and *Chrysosplenium* are consistent with a lack of signs of relaxed selection (Scobeyeva *et al.*, 2021; T. Yang *et al.*, 2023). However, in Lentibulariaceae, significant relaxed purifying selection was detected in some (but not all) *ndh* genes (Wicke *et al.*, 2014), and some (but not all) autotrophic species of Santalales showed relaxed selection of the combined group of *ndh* genes (Edlund *et al.*, 2025). Thus, current evidence does not support a general evolutionary scenario prior to loss of the *ndh* genes.

With regard to structural changes, complete loss of *ndh* genes obviously shortens the plastome; in particular, the short single copy (SSC) region housing 7 of the 11 *ndh* genes in canonical land plant plastomes (Ruhlman and Jansen, 2018). One of the genes, *ndhF*, is often located close to or at the border between the SSC and the IR_B_ region, and minor border shifts are frequently observed in taxa with *ndhF* gene loss or pseudogenization (e.g. H. T. Kim *et al.*, 2015, 2020; Könyves *et al.*, 2021). Numerous studies, some of which are cited below, have addressed a possible association between loss of *ndh* genes and more drastic plastome alterations, including expansion, contraction or even complete loss of the IR region. In an early study, Sanderson *et al.* (2015) found a statistically significant association between *ndh* gene loss and IR loss across land plants, although they acknowledged limitations in the analysis.

With more data at hand, the picture has not become clearer. A strong association between *ndh* loss and major IR contraction can be observed within the Zygophyllaceae (L. Zhang *et al.*, 2021; X. Wang *et al.*, 2022), but many other studied groups appear to lack any clear correlation, e.g. *Selaginella* (X.-M. Zhou *et al.*, 2022), Bromeliaceae (Ramírez-Morillo *et al.*, 2024), *Corydalis* (Raman *et al.*, 2022; S.-C. Kim *et al.*, 2023), Circaeasteraceae (Y. Sun *et al.*, 2017, 2020) and Plantaginaceae (Mower *et al.*, 2021). Depending on the phylogeny of the gymnosperms, loss of *ndh* genes might be a synapomorphy for Pinaceae and the gnetophytes, but only Pinaceae have strongly modified and very short IRs, a characteristic shared with cupressophytes having retained a normal *ndh* gene complement (C.-P. Lin *et al.*, 2010; Lubna *et al.*, 2021). An exception among the cupressophytes is the only gymnosperm heterotroph, *Parasitaxus*, that has lost the *ndh* genes but regained an IR (Qu *et al.*, 2019). Thus, no obvious association between *ndh* loss and structural changes exists for the gymnosperms, and persistent phylogenetic ambiguity prevents conclusive establishment of the order of key evolutionary events.

In the Cactaceae, the evolutionary scenario is also elusive. One clade, comprising the subfamily Cactoideae, is characterized by a common loss of *ndh* genes and some species within the clade also by IR modifications or even loss (Köhler *et al.*, 2023). However, in another clade, comprising subfamily Opuntioideae, where IR modifications and loss are abundant, only a small subclade has experienced *ndh* loss (Jie Yu *et al.*, 2023). Thus, again it cannot be postulated that either of the two types of events facilitates the other.

In another angiosperm group, Gentianaceae, where *ndh* loss has occurred repeatedly, three of five events of loss are associated with either IR expansion or contraction, thus here it can be suggested that *ndh* loss might facilitate IR border modifications (P.-C. Fu *et al.*, 2021). In contrast, the opposite scenario was suggested for the genus *Erodium* (Blazier *et al.*, 2016). Here, loss of *ndh* genes and loss of the IR are confined to different species, but it is argued that IR loss occurred ancestrally, but was followed by a regain in a clade of species exhibiting *ndh* loss (Blazier *et al.*, 2016).

Thus, in many groups of plants, *ndh* gene loss does go hand in hand with structural alterations of the plastome, but in others not. It remains possible that structural modifications, such as IR loss, can cause gene loss, or vice versa. However, it is clear that no general pattern exists.

## NDH LOSS AND EXTINCTION

Given that the function of NDH has been associated with alleviation of oxidative stress and changing environmental conditions, it has been assumed that genes over evolutionary time will be retained in periods of abiotic stress (Ruhlman *et al.*, 2015), whereas what has been referred to as ‘mild environments’ would render the genes dispensable and favour their deletion (Martín and Sabater, 2010; Sabater, 2021). However, subsequent to functional loss, Sabater also advocated that changing environments would decrease the viability and eventually drive plants without a functional NDH complex towards extinction, thus ‘they are evolutionary endpoints in phylogenetic trees’ (Sabater, 2021: p. 1).

These thoughts were picked up by Y. Yang *et al.* (2022), who suggested that *Tetraena mongolica* would face an extinction risk, in particular with changes in the environment. Likewise in Circaeasteraceae, *Kingdonia uniflora* was suggested to owe its status as endangered (in comparison to its more widespread sister species, *Circaeaster agrestis*) to a combination of environmental changes and NDH loss (Y. Sun *et al.*, 2020), and similar speculations were made about another endangered species, *Saniculiphyllum guangxiense* (Folk *et al.*, 2020). For jojoba (*Simmondsia chinensis*), it was even proposed that the monotypic genus *Simmondsia* now includes only one species owing to the extinction of others following the loss of NDH and lack of ability to survive in ‘a harsher environment’ (Kharabian-Masouleh *et al.*, 2023: p. 5024). Related, but partly reversed, thoughts were expressed for *Mikania* (Asteraceae), where the invasive habit of *M. micrantha* was speculated to be associated with a normal *ndh* gene complement, in comparison to the pseudogenization of *ndhF* in the non-invasive *M. cordata* (Y. Su *et al.*, 2018).

However, as evidence of gene loss accumulates, nothing suggests that plants without a functional NDH complex are more prone to extinction, and generally they are not more rare, endangered or non-invasive than other plants. In the genus *Allium*, NDH loss is so far restricted to the widespread species *A. paradoxum*, even considered an invasive weed (Omelchenko *et al.*, 2020). Although it has not been tested formally, there is no evidence that the pattern of speciation is affected within larger genera, such as *Corydalis* and *Gentiana*, where NDH loss has occurred repeatedly (P.-C. Fu *et al.*, 2021, 2022; S.-C. Kim *et al.*, 2023). In Zygophyllaceae, *Tetraena mongolica* might be endangered, but NDH loss most probably applies to the entire widespread subfamily Zygophylloideae, barely on the brink to extinction. The same will apply to the Cactaceae, where many species are endangered, but barely because of the loss of NDH, which probably characterize the entire subfamily Cactoideae plus some Opuntioideae (Köhler *et al.*, 2023; Jie Yu *et al.*, 2023). Within ancient clades, such as the gnetophytes and Pinaceae, where all species are thought to lack NDH, there are also rare and endangered species, but the clades have proved their resilience to change over extended evolutionary time.

## CONCLUSIONS AND FUTURE PROSPECTS

As an increasing number of plastome sequences become available, an increasing number of independent losses of *ndh* genes are discovered. Currently, >100 independent events of loss have been reported from autotrophic land plants. Data from nuclear genomes strongly suggest that these losses are coupled with the complete loss of the entire NDH complex. However, why the NDH complex is lost in some lineages and retained in others remains unclear. Loss in all but one clade of parasitic plants and in all investigated mycoheterotrophic lineages cannot be a coincidence; nor can a similar loss in half or more of the clades of carnivorous plants. These losses not only demonstrate that NDH is not needed in non-photosynthetic plants, but they also suggest a general relaxed need for NDH in plants relying on alternative sources of nutrition. However, the identification of an increasing number of autotrophic plant lineages also having lost *ndh* genes (and probably complete NDH) is enigmatic. Are all these plants relying on mycorrhizal partners to an extent rendering NDH superfluous? Is there a more fundamental cause that can explain losses, or are losses caused by different factors in different lineages? Maybe losses are random, and the affected plants are simply capable of survival without the NDH complex.

NDH is one of two alternative pathways of CET, and in model plants, NDH has been shown to be dispensable in controlled environments (Shikanai, 2020). As suggested previously, loss of *ndh* genes could be a step in the ongoing process of streamlining the plastome (C.-S. Wu *et al.*, 2009; Sanderson *et al.*, 2015), albeit not by functionally transferring the genes to the nuclear genome, but by deleting the NDH complex completely. Given that NDH is apparently dispensable in many diverse land plants, it has not only led to speculation regarding the reasons for gene loss, but the reverse question of why the genes are retained in most plants has also been asked (Wickett *et al.*, 2008*b*). Looking for a cause of either loss or retention might be two sides of the same coin, and the overarching problem in understanding the dependence or dispensability of NDH remains our lack of full knowledge about the role of NDH in photosynthesis or other metabolic pathways. Thus, further physiological and genetic research is severely needed.

Also needed are thorough studies investigating the potential correlation between presence/absence of genes and relevant environmental or biological factors. The current literature is full of speculation, but largely devoid of formal statistical testing. In trying to correlate NDH loss with any other parameter, it can be important to identify the first events of pseudogenization or to find patterns of significantly relaxed selection of NDH genes in close relatives with full, functional gene complements. The reason for NDH loss, if any beyond random, might lie prior to observable pseudogenization. In this context, relevant data are sparse, and although some studies of potential relaxed selection of plastid *ndh* genes have been made, they do not give a clear, consistent answer (Lallemand *et al.*, 2019; Edlund *et al.*, 2025). Furthermore, it is not known whether pseudogenization begins in the nuclear or plastid genome, or even if there is a general order.

In the present review, evidence from complete plastome sequences available in GenBank that are not accompanied by peer-reviewed publications has not been considered. As discussed previously, even reference sequence plastome sequences can contain errors, such as missing or wrong annotations, rendering manual curation necessary (Tonti-Filippini *et al.*, 2017). Although misannotation can also apply to sequences used in peer-reviewed publications, it can be assumed that authors actively writing about certain genes, such as *ndh* genes, are more likely to annotate them correctly. However, GenBank sequences lacking a publication might still be a source for discovery of new potential cases of *ndh* loss worthy of further exploration, and sequence annotation indicating multiple losses or pseudogenes are most likely to be correct. But in order to find candidates for studies of early stage NDH loss, it could be tempting to mine for plastome sequences lacking a single gene or having a single *ndh* pseudogene annotation. However, among such sequences, the risk for misannotation (or sequencing or assembly error) can be significant.
